# Fecal calprotectin as a marker of gastrointestinal involvement in pediatric Henoch–Schönlein purpura patients: a retrospective analysis

**DOI:** 10.1186/s12887-020-02263-x

**Published:** 2020-08-08

**Authors:** Eun Young Paek, Dae Yong Yi, Ben Kang, Byung-Ho Choe

**Affiliations:** 1 Department of Pediatrics, Chung-Ang University Hospital, 102, Heukseok-ro, Dongjak-gu, Seoul, 06973 Republic of Korea; 2 College of Medicine, Chung-Ang University, Seoul, South Korea; 3 Department of Pediatrics, School of Medicine, Kyungpook National University, Daegu, South Korea

**Keywords:** Gastrointestinal tract, Child, Calprotectin, Henoch–Schönlein purpura

## Abstract

**Background:**

Henoch–Schönlein purpura is a type of systemic vasculitis found in children. Its prognosis is usually good; however, recurrence is relatively common. If the intestines are affected, severe complications could arise. Here, we investigated the value of fecal calprotectin in the early screening of Henoch–Schönlein purpura and as a useful factor for predicting gastrointestinal manifestations.

**Methods:**

We retrospectively reviewed the medical records of pediatric patients who were diagnosed with Henoch–Schönlein purpura and underwent fecal calprotectin testing during the acute phase. The patients were categorized into gastrointestinal involvement and non-gastrointestinal involvement groups based on their clinical symptoms. Moreover, gastrointestinal involvement was categorized as follows: upper gastrointestinal tract involvement (up to the duodenum) and lower gastrointestinal tract involvement (from the terminal ileum).

**Results:**

A total of 69 patients were diagnosed with Henoch–Schönlein purpura and underwent fecal calprotectin testing. Among them, 40 patients (58.0%) showed signs of gastrointestinal involvement. The gastrointestinal involvement group had higher fecal calprotectin levels (379.9 ± 399.8 vs. 77.4 ± 97.6 mg/kg, *P* = 0.000). There were no significant differences in the recurrence of Henoch–Schönlein purpura symptoms or gastrointestinal symptoms. The cut-off value to identify gastrointestinal involvement was 69.10 mg/kg (*P* < 0.01). Patients with fecal calprotectin levels of > 50 mg/kg showed more frequent gastrointestinal involvement (77.8% vs. 20.8%, *P* = 0.000) and more severe gastrointestinal symptoms. Significant differences in abdominal pain duration, Henoch–Schönlein purpura clinical score, and abdominal pain severity were observed (*P* = 0.002, *P* = 0.000, and *P* = 0.000, respectively). Additionally, fecal calprotectin levels were significantly higher in patients with lower gastrointestinal tract involvement (214.67 ± 150.5 vs. 581.8 ± 510.1 mg/kg, *P* = 0.008), and the cut-off value was 277.5 mg/kg (*P* < 0.01).

**Conclusion:**

Fecal calprotectin testing is useful for identifying gastrointestinal involvement in pediatric Henoch–Schönlein purpura patients.

## Background

Henoch–Schönlein purpura is a type of systemic vasculitis involving small vessels, which is characterized by purpuric skin rashes, arthritis, and gastrointestinal and renal symptoms [1]. The prognosis of Henoch–Schönlein purpura is usually good; however, recurrence is relatively common. If the intestines or kidneys are affected, treatment may be needed [2]. The patient’s intestines are affected in approximately 30% of all cases, and without appropriate treatment, it can lead to severe complications including intussusception or intestinal perforation [3]. Abdominal pain, vomiting, melena, hematochezia, and other gastrointestinal symptoms may indicate gastrointestinal involvement in Henoch–Schönlein purpura patients; however, the symptoms may be nonspecific. Furthermore, if the symptoms are not severe, it is challenging to make an early clinical diagnosis [4].

Recently, certain laboratory markers or clinical scoring systems have been used to determine the diagnosis or severity of Henoch–Schönlein purpura. Moreover, esophagogastroduodenoscopy and imaging techniques such as abdominal ultrasonography and computed tomography have been used to confirm the severity of gastrointestinal involvement [5]. However, the existing diagnostic methods have limitations in terms of early diagnosis or accuracy and are invasive or inconvenient for follow-up examinations.

Calprotectin is a substance predominantly found in neutrophilic granulocytes, and its levels may be elevated under inflammatory conditions. Calprotectin can be detected in feces because neutrophils migrate to the intestinal mucosa when intestinal inflammation occurs. Therefore, fecal calprotectin elevation indicates intestinal inflammation, and it has been used as a biomarker for diagnosing inflammatory bowel disease [6]. Furthermore, it can be used as an indicator of other intestinal diseases, including polyps and necrotizing enterocolitis [7]. We hypothesized that fecal calprotectin levels may be used to determine gastrointestinal involvement, severity, recurrence, and invasion in Henoch–Schönlein purpura patients. Here, we investigated the value of fecal calprotectin in the early screening and diagnosis of complications for Henoch–Schönlein purpura patients and its usefulness for predicting gastrointestinal manifestations.

## Methods

### Patient selection

We retrospectively reviewed the medical records of pediatric patients aged < 18 years who were diagnosed with Henoch–Schönlein purpura and hospitalized at Chung-Ang University Hospital or Kyungpook National University Hospital between February 2015 and June 2019. All Henoch–Schönlein purpura patients were diagnosed according to the EULAR/PRINTO/PRES criteria. Among these patients, those who underwent fecal calprotectin testing during the acute phase were selected as the study group. Cases of an uncertain Henoch–Schönlein purpura diagnosis and cases showing comorbidities, such as a positive stool culture result (which may affect fecal calprotectin levels), were excluded from this study. Additionally, patients with indefinite gastrointestinal symptoms and patients with abdominal pain that could have been caused by other factors, including fecal impaction, were excluded. Consequently, 69 patients were included in the study, and their clinical information, laboratory results, ultrasonography results, and computed tomography results were reviewed.

### Data extraction

The patients were categorized into gastrointestinal involvement and non-gastrointestinal involvement groups based on clinical symptoms, including abdominal pain, vomiting, hematemesis, melena, and abdominal tenderness. In addition to the clinical symptoms, the results of esophagogastroduodenoscopy and imaging techniques were used to confirm gastrointestinal involvement and to determine the level of involvement. Gastrointestinal involvement was categorized according to the two most frequently involved sites as follows: upper gastrointestinal tract involvement (up to the duodenum) and lower gastrointestinal tract involvement (from the terminal ileum) [3, 4]. Medical records were reviewed to investigate the duration of hospitalization, joint or kidney involvement, and symptom recurrence, and the clinical scores of Henoch–Schönlein purpura and severity of gastrointestinal involvement were determined (Table 1) [8, 9]. The scores for the skin were not calculated because scoring was difficult based on medical records alone. Laboratory findings, including white blood cell count, absolute neutrophil count, erythrocyte sedimentation rate, C-reactive protein level, fibrin degradation product (FDP) level, D-dimer level, and stool occult blood status were also assessed [10, 11]. FDP and D-dimer tests were only performed at Chung-Ang University Hospital and not Kyungpook National University Hospital. Fecal calprotectin (GEMINI, Strategic Biomedical, Darmstadt, Germany) tests were performed on the first day of hospitalization, and the results were measured up to 2000 mg/kg at both hospitals. All patients were divided into two groups based on a fecal calprotectin level of 50 mg/kg, which is considered as the normal threshold for children aged > 4 years, and intergroup comparisons were performed [12].

**Table 1:** Clinical scoring of children with Henoch–Schönlein purpura [8, 9]

Organ involvement	Clinical score
Joint	0 = no symptoms
	1 = mild grade of pain and/or joint swelling
	2 = moderate grade of pain and/or joint swelling
	3 = severe grade of pain and/or joint swelling
Abdomen	0 = no symptoms
	1 = mild abdominal pain and/or stool occult blood (1+)
	2 = moderate abdominal pain and/or stool occult blood (2+/3+)
	3 = severe abdominal pain and/or melena
Kidney	0 = no proteinuria and no hematuria
	1 = proteinuria (1+) and/or hematuria (1+)
	2 = proteinuria (2+/3+) and/or hematuria (2+/3+)
	3 = proteinuria (> 3+) and/or hematuria (> 3+)

### Ethics statement

This study was conducted after obtaining approval from the Institutional Review Board of Chung-Ang University Hospital (IRB no. 1712–014-16,123) and Kyungpook National University Hospital (IRB no. 2020–03-019), and informed consent was waived owing to the retrospective nature of the study.

### Statistical analysis

Statistical analysis was performed using SPSS 18.0 statistical software (SPSS Inc., Chicago, IL, USA). The chi-square test and Student’s t-test were used to compare groups, and the corresponding data are presented as the mean and standard deviation. The linear regression test was used to compare continuous variables such as abdominal pain duration, Henoch–Schönlein purpura clinical score, and abdominal pain severity. ROC curves were used to determine the diagnostic cut-off value of the fecal calprotectin level to identify gastrointestinal involvement and the involved sites in the gastrointestinal tract. *P* values of < 0.05 were considered to be statistically significant.

## Results

### Clinical features and laboratory results of pediatric Henoch–Schönlein purpura patients who underwent fecal calprotectin testing

A total of 69 patients were diagnosed with Henoch–Schönlein purpura and underwent fecal calprotectin testing in both hospitals (57 patients at Chung-Ang University Hospital and 12 patients at Kyungpook National University Hospital) (Table 2). Of the 69 patients, 37 patients (53.6%) were male, and 32 patients (46.4%) were female; their mean age was 6.85 ± 2.93 years. Among the patients, 40 patients (58.0%) showed signs of gastrointestinal involvement, including vomiting, abdominal pain, bloody stool, or significant test results from imaging studies, and 29 patients (42.0%) showed no evidence of gastrointestinal involvement. Overall, 23 patients (33.3%) showed recurring symptoms involving the skin, joints, and gastrointestinal tract, and 19 patients (27.5%) showed recurring gastrointestinal symptoms unrelated to the initial ones.

**Table 2:** Clinical manifestations and laboratory results of Henoch–Schönlein purpura patients with and without gastrointestinal involvement

Variable	Total HSP patients (*n* = 69)	Gastrointestinal involvement (*n* = 40)	Non-gastrointestinal involvement (*n* = 29)	*P* value
Age (years)	6.85 ± 2.93	7.11 ± 2.67	6.48 ± 3.27	0.380
Male sex	37 (53.6%)	21 (52.5%)	16 (55.2%)	0.826
Duration of admission (days)	5.41 ± 5.27	7.25 ± 6.05	2.79 ± 1.97	0.000*
Joint symptoms	35 (50.7%)	16 (40.0%)	19 (65.5%)	0.036*
Kidney involvement	16 (23.2%)	10 (25.0%)	6 (20.7%)	0.675
HSP clinical score	1.99 ± 1.53	2.65 ± 1.59	1.07 ± 0.80	0.000*
HSP recurrence	23 (33.3%)	15 (37.5%)	8 (27.6%)	0.389
Gastrointestinal symptom recurrence	19 (27.5%)	12 (30.0%)	7 (24.1%)	0.591
Positive stool occult blood result	17 (24.6%)	17 (42.5%)	0 (0%)	0.000*
Fecal calprotectin (mg/kg)	252.7 ± 343.8	379.9 ± 399.8	77.4 ± 97.6	0.000*
WBC count (10^6^/L)	11,757 ± 5133	12,365 ± 6015	10,889 ± 3434	0.246
ANC (μL)	7673 ± 4388	8476 ± 4716	6526 ± 3652	0.071
ESR (mm/h)	23.74 ± 12.46	25.50 ± 12.62	21.04 ± 11.95	0.157
C-reactive protein (mg/L)	5.87 ± 6.39	5.18 ± 5.65	6.87 ± 7.31	0.285
FDP (μg/mL) (*n* = 57)	9.14 ± 9.16	11.08 ± 11.14	6.73 ± 5.07	0.064
D-dimer (μg/mL) (*n* = 57)	2.34 ± 2.45	2.93 ± 2.89	1.61 ± 1.50	0.036*

*HSP* Henoch–Schönlein purpura, *WBC* White blood cell, *ANC* Absolute neutrophil count, *ESR* Erythrocyte sedimentation rate, *FDP* Fibrin degradation product

*Significant findings at *P* < 0.05

### Differences according to gastrointestinal involvement

**Fig. 1:**
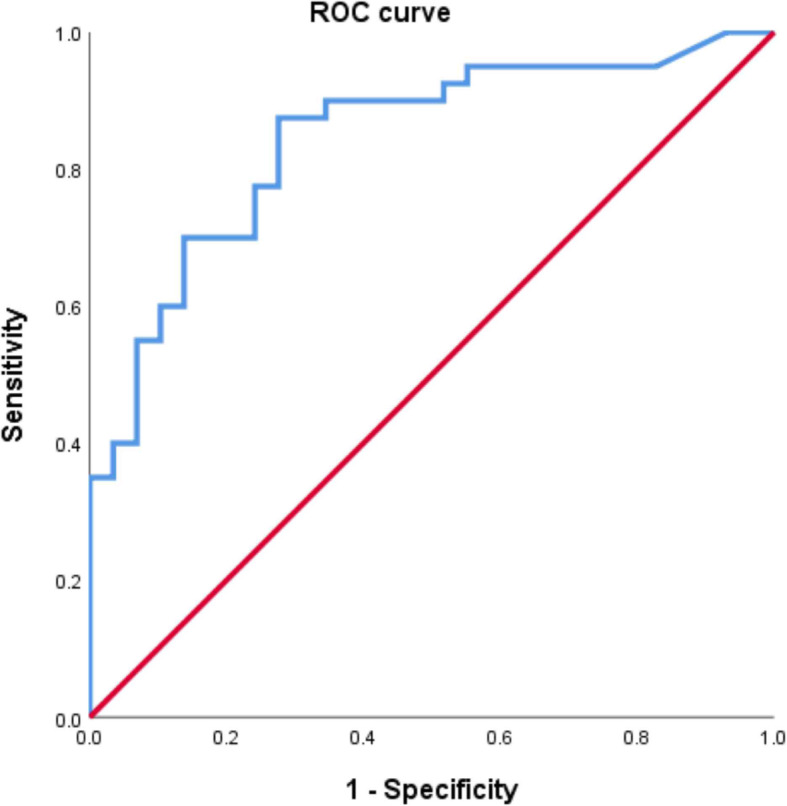
ROC curve of the fecal calprotectin level for diagnosing gastrointestinal involvement in pediatric Henoch–Schönlein purpura patients

The clinical manifestations of the gastrointestinal involvement group and the non-gastrointestinal involvement group were compared (Table 2). There was no difference in the mean age or sex between the two groups; however, the mean hospitalization period was longer in the gastrointestinal involvement group (7.25 ± 6.05 vs. 2.79 ± 1.97 days, *P* = 0.000). Patients with gastrointestinal involvement exhibited less frequent joint symptoms (40.0% vs. 65.5%, *P* = 0.036); however, kidney involvement was not significantly different. The gastrointestinal involvement group showed higher Henoch–Schönlein purpura clinical scores (2.65 ± 1.59 vs. 1.07 ± 0.80, *P* = 0.000) and higher fecal calprotectin levels (379.9 ± 399.8 vs. 77.4 ± 97.6 mg/kg, *P* = 0.000). There were no significant differences with regard to the recurrence of Henoch–Schönlein purpura or gastrointestinal symptoms. The area under the ROC curve for the fecal calprotectin level for detecting gastrointestinal involvement was 0.844, which was statistically significant (*P* < 0.01). The diagnostic sensitivity and specificity were 87.5 and 72.4%, respectively, when the cut-off value of the fecal calprotectin level was 69.10 mg/kg (Fig. 1). Additionally, stool occult blood status, FDP level, and D-dimer level were different between the two groups (*P* = 0.000, *P* = 0.064, and *P* = 0.036, respectively).

### Differences according to fecal calprotectin levels

The clinical manifestations of patients with fecal calprotectin levels over and under 50 mg/kg were compared (Table 3). Patients with fecal calprotectin levels of > 50 mg/kg showed more frequent gastrointestinal involvement (77.8% vs. 20.8%, *P* = 0.000), longer hospitalization duration (*P* = 0.043), and longer gastrointestinal symptom duration (*P* = 0.001). Moreover, Henoch–Schönlein purpura clinical scores and gastrointestinal symptom severity were also different (*P* = 0.005 and *P* = 0.000, respectively). However, the involved site, recurrence of Henoch–Schönlein purpura, or recurrence of gastrointestinal symptoms were not different.

**Table 3:** Clinical manifestations of Henoch–Schönlein purpura patients with increased fecal calprotectin levels

Variable	Total HSP patients (*n* = 69)	Fecal calprotectin > 50 mg/kg (*n* = 45)	Fecal calprotectin < 50 mg/kg (*n* = 24)	*P* value
Duration of admission (days)	5.41 ± 5.27	6.33 ± 5.88	3.61 ± 3.22	0.043*
Patients with gastrointestinal symptoms	40 (58.0%)	35 (77.8%)	5 (20.8%)	0.000*
Upper gastrointestinal tract involvement	22/40 (55.0%)	19/35 (54.3%)	3/5 (60.0%)	1.000
Lower gastrointestinal tract involvement	18/40 (45.0%)	16/35 (45.7%)	2/5 (40.0%)	
Duration of gastrointestinal symptoms (days)	2.88 ± 4.02	3.89 ± 4.35	1.00 ± 2.41	0.001*
HSP clinical score	1.99 ± 1.53	2.36 ± 1.61	1.29 ± 1.08	0.005*
Gastrointestinal involvement score	0.93 ± 0.93	1.27 ± 0.89	0.29 ± 0.62	0.000*
HSP recurrence	23 (33.3%)	18 (40.0%)	5 (20.8%)	0.108
Gastrointestinal symptom recurrence	19 (27.5%)	15 (33.3%)	4 (16.7%)	0.167
Positive stool occult blood result	17 (24.6%)	16 (35.6%)	1 (4.2%)	0.003*

*HSP* Henoch–Schönlein purpura

*Significant findings at *P* < 0.05

**Fig. 2:**
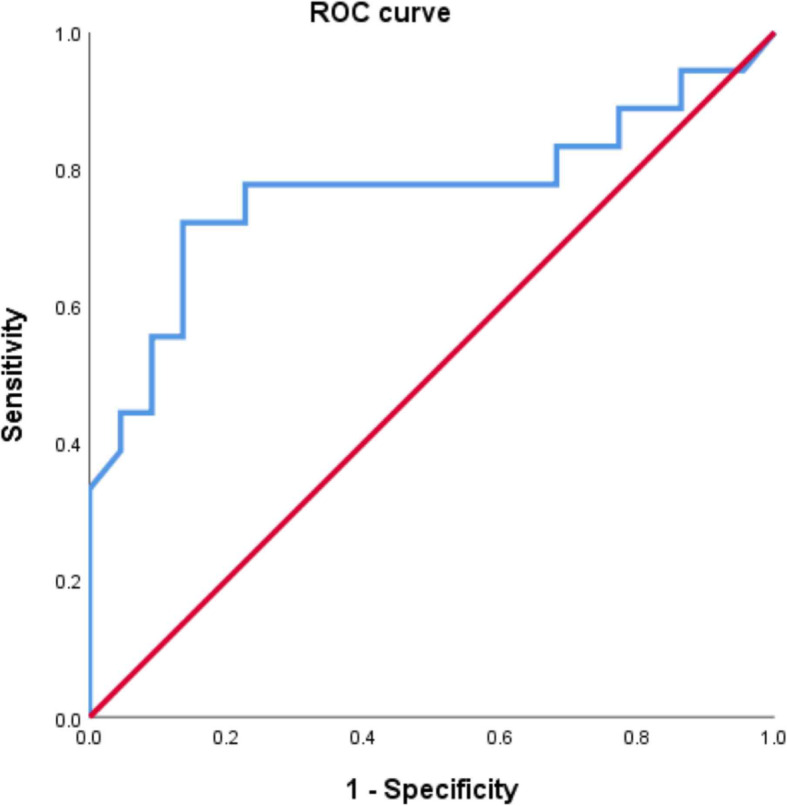
ROC curve of the fecal calprotectin level for diagnosing lower gastrointestinal involvement in pediatric Henoch–Schönlein purpura patients

As shown in Table 4, no significant differences in fecal calprotectin levels were observed when compared in terms of Henoch–Schönlein purpura recurrence and gastrointestinal symptom recurrence (*P* = 0.218 and *P* = 0.176, respectively). However, fecal calprotectin levels were significantly higher in patients with lower gastrointestinal tract involvement (from the terminal ileum) than in patients with upper gastrointestinal tract involvement (up to the duodenum) (214.67 ± 150.5 vs. 581.8 ± 510.1 mg/kg, *P* = 0.008). Additionally, significant differences were noted with regard to abdominal pain duration, Henoch–Schönlein purpura clinical score, and abdominal pain severity (*P* = 0.002, *P* = 0.000, and *P* = 0.000, respectively). The area under the ROC curve for the fecal calprotectin level for detecting lower gastrointestinal involvement was 0.768, which was statistically significant (*P* < 0.01). The diagnostic sensitivity and specificity were 77.8 and 77.3%, respectively, when the cut-off value of the fecal calprotectin level for detecting lower gastrointestinal involvement was 277.5 mg/kg (Fig. 2). The fecal calprotectin levels of Henoch–Schönlein purpura patients were not associated with other inflammatory markers including white blood cell count, absolute neutrophil count, erythrocyte sedimentation rate, C-reactive protein level, FDP level, and D-dimer level but were associated with stool occult blood status.

**Table 4:** Fecal calprotectin levels of Henoch–Schönlein purpura patients different clinical manifestations

Variable	Fecal calprotectin (mg/kg)		*P* value
Gastrointestinal involvement (no: yes)	77.4 ± 97.6	379.9 ± 399.8	0.000*
Site of gastrointestinal involvement (upper: lower)	214.7 ± 150.5	581.8 ± 510.1	0.008*
HSP recurrence (no: yes)	216.5 ± 361.5	325.3 ± 299.8	0.218
Gastrointestinal symptom recurrence (no: yes)	218.1 ± 349.4	344.0 ± 319.5	0.176
Stool occult blood result (negative: positive)	149.3 ± 170.4	569.3 ± 518.2	0.004*

*HSP* Henoch–Schönlein purpura

*Significant findings at *P* < 0.05

### Differences according to joint or kidney involvement

We compared fecal calprotectin results according to joint involvement and kidney involvement in Henoch–Schönlein purpura patients. There were no significant differences in fecal calprotectin levels between 35 patients with joint symptoms and 34 patients without symptoms (243.2 ± 378.5 vs. 262.6 ± 309.6 mg/kg, *P* = 0.817). In addition, the levels of inflammatory markers such as FDP and D-dimer did not differ between the two groups (10.01 ± 11.67 vs. 8.28 ± 5.78 μg/mL, *P* = 0.493 and 2.69 ± 3.05 vs. 1.99 ± 1.64, μg/mL *P* = 0.304, respectively). There were no significant differences in fecal calprotectin (317.1 ± 411.7 vs. 233.3 ± 322.5 mg/kg, *P* = 0.397), FDP (9.67 ± 9.91 vs. 6.85 ± 4.20 μg/mL, *P* = 0.385) and D-dimer (2.52 ± 2.63 vs. 1.56 ± 1.26 μg/mL, *P* = 0.267) levels between patients with kidney involvement (*n* = 16) and those without kidney involvement (*n* = 53).

## Discussion

Calprotectin is a 36.5 kDa long calcium- and zinc-binding protein consisting of heterodimers of the S100A8 (MRP-8) and S100A9 (MRP-14) subunits [13–15]. In particular, fecal calprotectin remains stable in feces for more than a week and thus is a useful marker for intestinal inflammatory reactions, and it is gradually being used to diagnose other intestinal diseases [16–19]. Other prospective studies have explored the association between fecal calprotectin and Henoch–Schönlein purpura [10, 20]. As these studies were prospective in nature, they were able to determine the changes in fecal calprotectin levels caused by improvements in Henoch–Schönlein purpura symptoms; in contrast, as our study had a retrospective design, we could not determine these changes.

In addition to demonstrating that fecal calprotectin is useful for detecting gastrointestinal invasion in Henoch–Schönlein purpura patients, our study provides some additional evidence. Previous studies compared gastrointestinal involvement in Henoch–Schönlein purpura patients with that in healthy controls, whereas in our study, all of the enrolled patients were diagnosed with Henoch–Schönlein purpura. Therefore, we could compare patients with gastrointestinal involvement and those without gastrointestinal involvement from a cohort of Henoch–Schönlein purpura patients in order to confirm the association of gastrointestinal involvement with fecal calprotectin. Furthermore, our results revealed that fecal calprotectin levels were dependent on the involved gastrointestinal site and were positively associated with Henoch–Schönlein purpura clinical scores including the severity of gastrointestinal symptoms. Kanik et al. previously reported that fecal calprotectin levels were associated with renal involvement [10]. Hence, we investigated the association of fecal calprotectin with kidney or joint involvement, which are the other major manifestations of Henoch–Schönlein purpura. However, no significant associations were confirmed in our results. Considering that fecal calprotectin is a marker of intestinal inflammation, there may be no association in cases with only kidney or joint involvement without gastrointestinal involvement. Furthermore, in another study by Teng et al., only the association of fecal calprotectin with gastrointestinal involvement was reported [20]. Finally, in our study, the cut-off fecal calprotectin level for identifying gastrointestinal involvement was 69.10 mg/kg, which was much lower than the value reported by Teng et al. (264.5 mg/kg) [20]. Interestingly, this value was similar to the cut-off level for identifying lower gastrointestinal involvement in our study (277.5 mg/kg). Fecal calprotectin is known as a specific marker for the diagnosis of colorectal inflammation, and Montalto et al. reported that its level did not increase during small bowel bacterial overgrowth [21, 22]. Another study showed that fecal calprotectin did not increase in chronic gastritis, even in patients with marked neutrophil infiltration [23]. Therefore, the higher level of fecal calprotectin observed in Henoch–Schönlein purpura patients with lower gastrointestinal tract involvement in our study may demonstrate the characteristics of fecal calprotectin. If fecal calprotectin is prospectively investigated according to the involved gastrointestinal site, our results may be validated.

In a study by Hong et al. [11], various inflammatory markers, including FDP and D-dimer, were present at significantly higher levels during the acute phase of Henoch–Schönlein purpura. However, in our study, only D-dimer was significantly increased in the presence of gastrointestinal involvement. FDP was also higher in patients with gastrointestinal symptoms; however, the result was not statistically significant. In a previous study, significant differences in D-dimer and FDP levels were noted among patients with more severe gastrointestinal symptoms. It was reported that a bidirectional relationship exists between the inflammation and coagulation systems. In the pathogenesis of vascular diseases such as Henoch–Schönlein purpura, the initiation of inflammation leads to the activation of the coagulation system, which also markedly affects inflammatory activity [24]. Despite the lack of the statistical significance observed in previous studies, our results may be explained by a similar pathogenesis.

The present study has some limitations. First, this study was a retrospective study; thus, the gastrointestinal involvement group was retrospectively classified based on clinical symptoms, physical examinations, and imaging test results. Patients who had abdominal pain likely caused by factors other than Henoch–Schönlein purpura were excluded from the study group; the underlying cause of the pain was confirmed via procedures such as ultrasonography and a combination of computed tomography and esophagogastroduodenoscopy for most patients, except for one patient with gastrointestinal symptoms. Nevertheless, there is still a possibility that these patients were included. Second, fecal calprotectin was obtained at the time of admission; however, there were cases in which fecal calprotectin was actually collected 2–3 days later because the patient did not defecate. Furthermore, fecal calprotectin testing was performed only for hospitalized patients and not for outpatient patients or patients with mild symptoms. Therefore, fecal calprotectin testing was not performed for all Henoch–Schönlein purpura patients during the study period. Finally, of the 23 relapsed patients, 19 patients had gastrointestinal symptom recurrence.

## Conclusions

Despite the limitations, this study demonstrated that fecal calprotectin might be useful for determining the involved site and course of gastrointestinal symptoms as well as predicting gastrointestinal involvement in pediatric Henoch–Schönlein purpura patients. Better results may be obtained if a larger number of patients are evaluated, including those with simple skin rashes only and those treated as outpatients.

## Data Availability

The data will not be shared because data will be used as material for other studies.

## References

[1] Jennette JC, Falk RJ, Bacon PA, Basu N, Cid MC, Ferrario F (2013). 2012 revised international Chapel Hill consensus conference nomenclature of vasculitides. Arthritis Rheum.

[2] Davin JC, Coppo R (2013). Pitfalls in recommending evidence-based guidelines for a protean disease like Henoch-Schönlein purpura nephritis. Pediatr Nephrol.

[3] Hwang HH, Lim IS, Choi BS, Yi DY (2018). Analysis of seasonal tendencies in pediatric Henoch-Schönlein purpura and comparison with outbreak of infectious diseases. Medicine (Baltimore).

[4] Reamy BV, Williams PM, Lindsay TJ (2009). Henoch-Schönlein purpura. Am Fam Physician.

[5] Schwab J, Benya E, Lin R, Majd K (2005). Contrast enema in children with Henoch-Schönlein purpura. J Pediatr Surg.

[6] Walsham NE, Sherwood RA (2016). Fecal calprotectin in inflammatory bowel disease. Clin Exp Gastroenterol.

[7] Park Y, Son M, Jekarl DW, Choi HY, Kim SY, Lee S (2019). Clinical significance of inflammatory biomarkers in acute pediatric diarrhea. Pediatr Gastroenterol Hepatol Nutr.

[8] De Mattia D, Penza R, Giordano P, Del Vecchio GC, Aceto G, Altomare M (1995). von Willebrand factor and factor XIII in children with Henoch-Schonlein purpura. Pediatr Nephrol.

[9] Yilmaz D, Kavakli K, Ozkayin N (2005). The elevated markers of hypercoagulability in children with Henoch-Schönlein purpura. Pediatr Hematol Oncol.

[10] Kanik A, Baran M, Ince FD, Cebeci O, Bozkurt M, Cavusoglu D (2015). Faecal calprotectin levels in children with Henoch-Schönlein purpura: is this a new marker for gastrointestinal involvement?. Eur J Gastroenterol Hepatol.

[11] Hong J, Yang HR (2015). Laboratory markers indicating gastrointestinal involvement of Henoch-Schönlein purpura in children. Pediatr Gastroenterol Hepatol Nutr..

[12] Jeoung SJ (2019). The role of fecal calprotectin in pediatric disease. Korean J Pediatr.

[13] Bonnín Tomàs A, Vila Vidal M, Rosell CA (2007). Fecal calprotectin as a biomarker to distinguish between organic and functional gastrointestinal disease. Rev Esp Enferm Dig.

[14] Ezri J, Nydegger A (2011). Pediatrics. Fecal calprotectin in children: use and interpretation. Rev Med Suisse.

[15] Rodrigo L (2007). Fecal calprotectin. Rev Esp Enferm Dig.

[16] Jang HJ, Park JH, Kim CS, Lee SL, Lee WM (2018). Amino acid-based formula in premature infants with feeding intolerance: comparison of fecal calprotectin level. Pediatr Gastroenterol Hepatol Nutr..

[17] Kim SY, Lee NM, Yun SW, Chae SA, Lim IS, Choi ES (2019). Influence of proton pump inhibitor therapy on intestinal inflammation assessed by fecal calprotectin in pediatric patients. Korean J Pediatr..

[18] Höög CM, Bark LÅ, Broström O, Sjöqvist U (2014). Capsule endoscopic findings correlate with fecal calprotectin and C-reactive protein in patients with suspected small-bowel Crohn’s disease. Scand J Gastroenterol.

[19] Olsen PA, Fossmark R, Qvigstad G (2015). Fecal calprotectin in patients with suspected small bowel disease—a selection tool for small bowel capsule endoscopy?. Scand J Gastroenterol.

[20] Teng X, Gao C, Sun M, Wu J (2018). Clinical significance of fecal calprotectin for the early diagnosis of abdominal type of Henoch–Schonlein purpura in children. Clin Rheumatol.

[21] Fagerberg UL, Loof L, Myrdal U, Hansson LO, Finkel Y (2005). Colorectal inflammation is well predicted by fecal calprotectin in children with gastrointestinal symptoms. J Pediatr Gastroenterol Nutr Actions.

[22] Montalto M, Santoro L, Dalvai S, Curigliano V, D'Onofrio F, Scarpellini E (2008). Fecal calprotectin concentrations in patients with small intestinal bacterial overgrowth. Dig Dis.

[23] Montalto M, Gallo A, Ianiro G, Santoro L, D'Onofrio F, Ricci R (2010). Can chronic gastritis cause an increase in fecal calprotectin concentrations?. World J Gastroenterol Actions.

[24] Levi M, van der Poll T (2005). Two-way interactions between inflammation and coagulation. Trends Cardiovasc Med.

